# The Role of Late-Acting Self-Incompatibility and Early-Acting Inbreeding Depression in Governing Female Fertility in Monkshood, *Aconitum kusnezoffii*

**DOI:** 10.1371/journal.pone.0047034

**Published:** 2012-10-09

**Authors:** Yi-Qi Hao, Xin-Feng Zhao, Deng-Ying She, Bing Xu, Da-Yong Zhang, Wan-Jin Liao

**Affiliations:** State Key Laboratory of Earth Surface Processes and Resource Ecology and MOE Key Laboratory for Biodiversity Science and Ecological Engineering, Beijing Normal University, Beijing, China; University of Oxford, United Kingdom

## Abstract

Reduced seed yields following self-pollination have repeatedly been observed, but the underlying mechanisms remain elusive when self-pollen tubes can readily grow into ovaries, because pre-, post-zygotic late-acting self-incompatibility (LSI), or early-acting inbreeding depression (ID) can induce self-sterility. The main objective of this study was to differentiate these processes in *Aconitum kusnezoffii*, a plant lacking stigmatic or stylar inhibition of self-pollination. We performed a hand-pollination experiment in a natural population of *A. kusnezoffii*, compared seed set among five pollination treatments, and evaluated the distribution of seed size and seed set. Embryonic development suggested fertilization following self-pollination. A partial pre-zygotic LSI was suggested to account for the reduced seed set by two lines of evidence. The seed set of chase-pollination treatment significantly exceeded that of self-pollination treatment, and the proportion of unfertilized ovules was the highest following self-pollination. Meanwhile, early-acting ID, rather than post-zygotic LSI, was suggested by the findings that the size of aborted selfed seeds varied continuously and widely; and the selfed seed set both exhibited a continuous distribution and positively correlated with the crossed seed set. These results indicated that the embryos were aborted at different stages due to the expression of many deleterious alleles throughout the genome during seed maturation. No signature of post-zygotic LSI was found. Both partial pre-zygotic LSI and early-acting ID contribute to the reduction in selfed seed set in *A. kusnezoffii*, with pre-zygotic LSI rejecting part of the self-pollen and early-acting ID aborting part of the self-fertilized seeds.

## Introduction

Reduced female fertility following self-pollination is generally attributed to either self-incompatibility or early-acting inbreeding depression (ID). Most self-incompatibility systems primarily function in a pre-zygotic manner via the failure of self-pollen to germinate and grow in the stigma or style [1], [2], [3]. However, for many plant species self-pollen tubes grow successfully in the styles and extend to the ovaries, thus implicating late-acting self-incompatibility (LSI) [4], [5], [6], [7], [8], [9], [10]. In such cases, the inhibition of self-pollen tubes could occur either pre-zygotically or post-zygotically, which is commonly thought to be controlled by a single locus [5], [9], [10], [11], [12]. In post-zygotic LSI, the rejection occurs shortly after double fertilization, with little development of the embryos and/or embryo sacs [9], [13], [14]. Early-acting ID acts post-zygotically and leads to the abortion of developing embryos that are homozygous for many deleterious recessive alleles throughout the genome [15], [16], [17].

Inbreeding depression is thought to be one of the important selective forces that govern the evolution of plant reproductive strategies [15], [18], [19], [20], [21], and self-incompatibility can evolve in response to cumulative inbreeding depression [15]. Pre- and/or post-zygotic LSI and early-acting ID may interact and have complex consequences, and play a key role in determining the evolution of plant mating systems [22], [23]. In a study of *Dipterocarpus tempehes*, all the three processes are present and make contributions to outcrossing (Kenta *et al*., 2002). Despite the great importance, pre- vs. post-zygotic LSI and LSI vs. early-acting ID, have not been well distinguished to date. Indeed, it has been difficult to differentiate pre- and post-zygotic LSI because the data on pollen tube growth and differences in seed set between self- and cross-pollinations could not indicate the presence of double fertilization. Consequently, some studies did not take post-zygotic LSI into account and used the term ‘ovarian self-incompatibility’ to describe the situation in which self- pollen tubes grow into ovaries but result in low seed set [24], [25]. When double fertilization does occur following self-pollination, it remains difficult to distinguish post-zygotic LSI from early-acting ID, because there are many difficulties in scoring embryo development and identifying the mechanisms for reduced self-fertility. So far, only a few studies provided concrete evidence for the occurrence of late-acting self-incompatibility [8], [9], [11], [12], [26] or early-acting ID [24], [27], [28] or both [14].

Based on the timing of self-pollen rejection, at least two strategies have been proposed to differentiate pre-zygotic LSI from post-zygotic LSI and early-acting ID. Sectioning methods characterize the histological aspects of ovule-seed development following self- and cross-pollination [9], [13], [14]. Difference in the proportion of unfertilized ovules and seed set among various pollen chase experiments [24], [27] could provide evidence for the presence or absence of self fertilization.

The differentiation between early-acting ID and LSI is mainly based on the number of loci involved. A continuous distribution with large variation of aborted seed size and seed set after self-pollination would be interpreted as early-acting ID since the abortion of selfed embryos was due to many deleterious recessive alleles expressed at different developmental stages. To the contrary, a clumped distribution with little variation would be interpreted as LSI [5], [12], [27], [29], [30]. Besides, the positive correlation between selfed and crossed seed sets also supports the presence of early-acting ID [24]. According to the inbreeding depression model, an individual with more loci containing deleterious alleles will reduce both selfed and crossed seed sets [24], and hence such positive correlations between selfed and crossed seed sets may suggest that the maternal deleterious alleles are being expressed in both the selfed and crossed progeny [17], [27].

Other criteria include the response of embryos to rescue in tissue culture [5], [9], [31], induced mutations [5], [32], and segregation within families for self-incompatibility alleles [5], [9], [12]. However, the above-mentioned five approaches are the most amenable to empirical testing in the field through hand-pollination experiments.

In a previous study of *Aconitum kusnezoffii* Reichb. (Ranunculaceae), we found that self-pollination produced fewer seeds, even though self-pollen tubes entered ovaries at a similar growth rate as cross-pollen tubes [33]. We tentatively concluded that early-acting ID, rather than LSI, most likely reduces female function within large clones, but lacked concrete evidence for fertilization and ID during seed maturation [33].

In this study, we aimed to ascertain whether pre- or post-zygotic LSI and/or early-acting ID reduce female reproductive success after self-pollination in *A. kusnezoffii*. We focused on two major questions: (1) does self-pollination lead to self-fertilization; and (2) is embryo abortion following self-pollination attributable to early-acting inbreeding depression or post-zygotic self-incompatibility? We performed self-, cross-, chase-, and mixed-pollinations in a single *A. kusnezoffii* population and used the five methods described above to distinguish between LSI and ID.

## Materials and Methods

### Ethics Statement

No specific permits were required for the described field studies, and the field studies did not involve endangered or protected species.

### Study Species and Sites

*Aconitum kusnezoffii* is a bumblebee-pollinated and predominantly outcrossing herb [33]. It grows clonally via tubers and thus has a clumped architecture. The flower is perfect, having 3–5 separated carpels. It flowers in August, with four to five days of pollen exposure, then about two days of stigma receptivity. The field work was conducted in a natural population of Xiaolongmen National Forest Park (39°57′32.1″N, 115°27′03.8″E, 1034 m elevation), West Beijing, China.

### Hand-pollination

To account for fine-scale environmental variation, we used a randomized block design, combining different pollination treatments in the same terminal inflorescence. We randomly selected 41 clones and a total of 107 terminal inflorescences. For each inflorescence, five hermaphroditic flowers were randomly assigned to one of the following five pollination treatments: (1) self-pollination, with pollen from other open flowers of the same clone; (2) cross-pollination, with pollen from other clones; (3) chase-pollination, with flowers being self-pollinated first and then cross-pollinated after 24 h; (4) mixed-pollination, with pollen from pollen grain mixtures of two anthers (one from the same clone and the other from other clones) and applied simultaneously; and (5) open-pollinated, with flowers left unmanipulated and open to natural pollination.

We emasculated and bagged all sampled flowers, except those exposed to open pollination. Once the stigmas of the four bagged flowers became receptive, the flowers were marked and hand-pollinated with the appropriate pollen. We removed the bags 3 days after hand-pollination and collected all ripe fruits 4 weeks later. Because of herbivore damage, we obtained only 76 inflorescences from the 32 clones at harvest.

We counted the mature seeds, aborted seeds and unfertilized ovules for each fruit from all of the five pollination treatments. Mature seeds were relatively large, plump, fresh, and green, whereas aborted seeds were shrunken, light-colored, and flat. Unfertilized ovules were extremely small and round, but still visible. We estimated seed set as the ratio of mature seeds to the sum of mature seeds, aborted seeds, and unfertilized ovules, i.e. the total number of available ovules within a fruit. We also measured the lengths of all aborted and mature seeds from 13 self-pollinated fruits and 13 cross-pollinated fruits on the same ramets to the nearest 0.01 mm; unfertilized ovules were too small to measure.

### Fertilization Following Self- and Cross-pollination

To compare the embryological development of the selfed seeds with crossed seeds, we emasculated and bagged another four flowers from two ramets, self-pollination performed on one ramet and cross-pollination performed on the other. One and three days following self- or cross-pollination, we collected one selfed and one crossed ovary, which were fixed in formalin-acetic acid-ethanol (FAA), and later washed and dehydrated by increasing gradients of alcohol. The ovaries were then infiltrated in dimethylbenzene, embedded in paraffin at a melting point of 60°C, sectioned and stained with eosin Y and fast green. The serial sections were scored using a light microscope for the occurrence of fertilization.

### Data Analysis

The seed sets following the five pollination treatments were analyzed with a linear mixed model in R statistical package [34]. The seed set were arcsin transformed to meet the assumptions of normality and homogeneity of variance. Pollination treatment was considered as the fixed factor, and clone and ramet were considered as random factors, with ramet nested within clone. The variance in the seed set following self- and cross-pollination among all 76 ramets was also estimated. The data of proportion of unfertilized ovules was not normally distributed, so the data was analyzed using paired Wilcoxon’s test in R [34]. Lastly, we estimated the linear correlation between self- and cross-seed sets at both the ramet and clone levels.

## Results and Discussion

### Pre- and/or Post-zygotic Process

**Figure 1:**
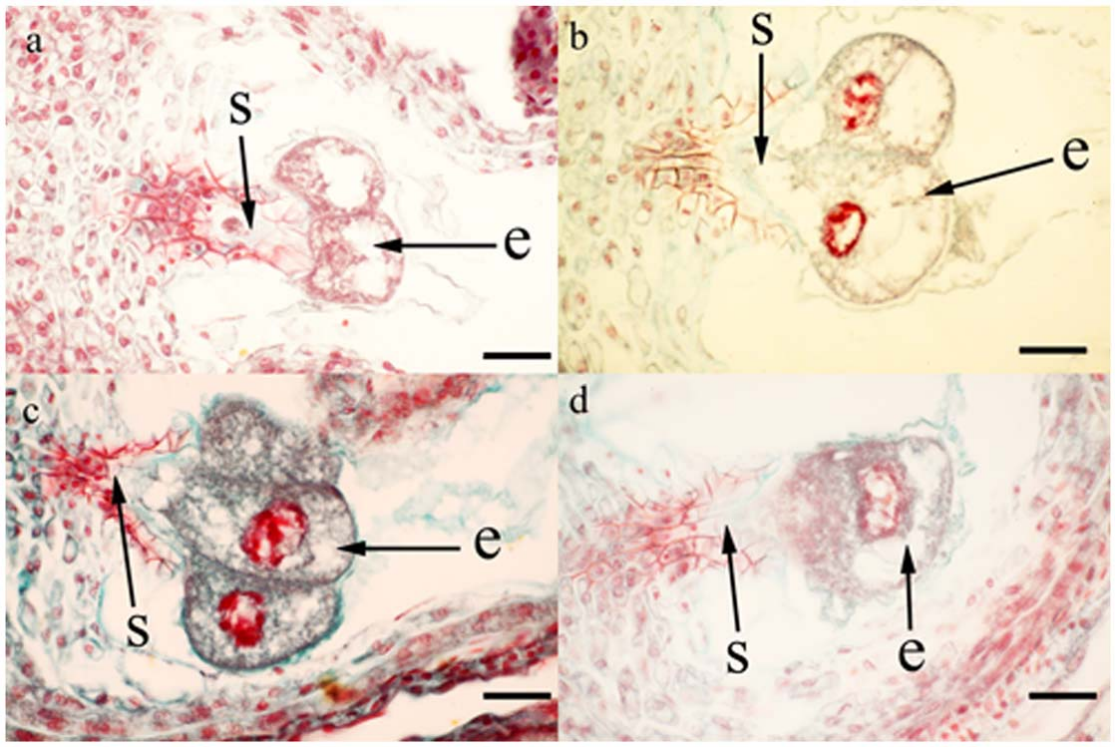
Light micrographs of serial sections of fertilized *Aconitum kusnezoffii* ovaries. (a ). ovary harvested one day after self-pollination. (b ). ovary harvested one day after cross-pollination. (c ). ovary harvested three days after self-pollination. (d ). ovary harvested three days after self-pollination. Abbreviations: s, suspensor; e, proembryo cell. Bar = 50 µm.

Serial sections of ovaries showed that zygotes have formed and begun to divide within 1 day after both self- and cross-pollination (Fig. 1). Suspensor and proembryo cells could be seen clearly, and there was no significant difference in the histological aspects of ovule-seed development following self- and cross-pollination. This provided direct evidence for the occurrence of fertilization following self- and cross-pollination.

**Figure 2:**
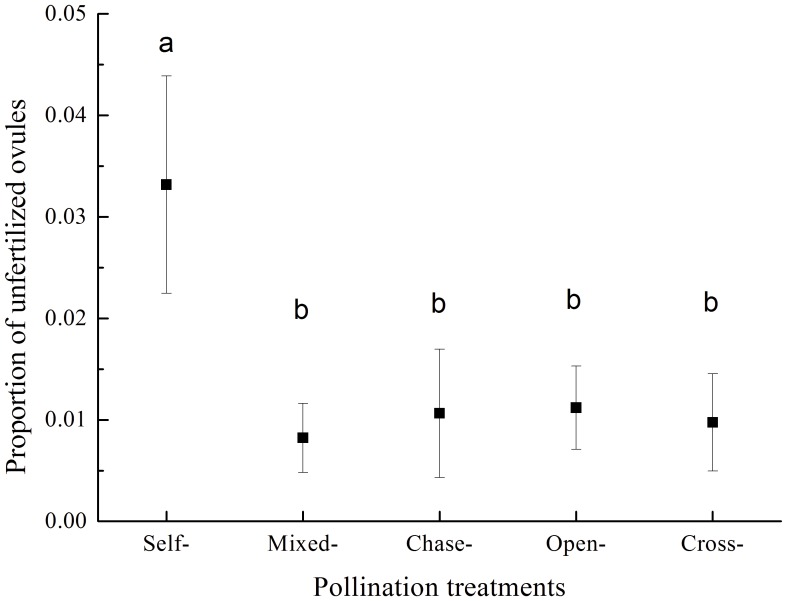
The mean (± SE ) proportion of unfertilized ovules in *Aconitum kusnezoffii* following five pollination treatments. Different lowercase letters indicate significant differences (*P*<0.05) in the proportion of unfertilized ovules.

**Figure 3:**
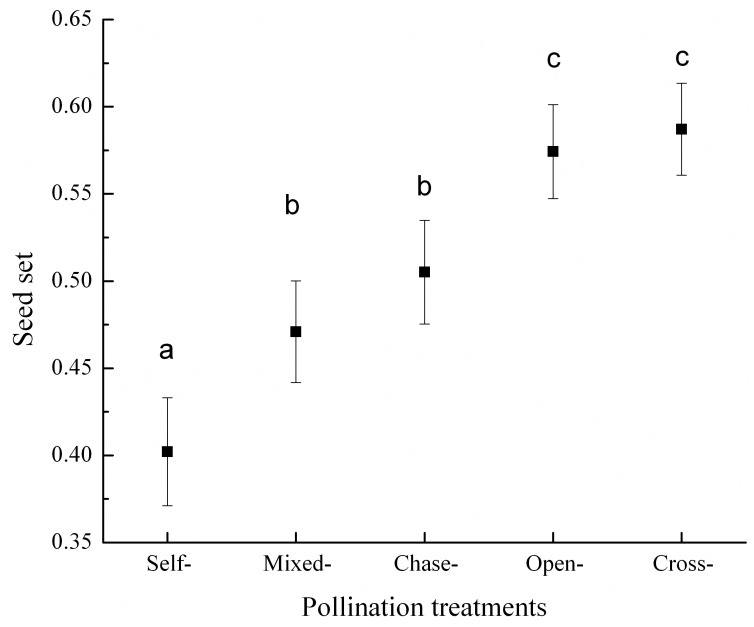
Mean (± SE) seed sets in *Aconitum kusnezoffii* following five pollination treatments. Different lowercase letters indicate significant differences (*P*<0.05) in the seed sets.

The proportion of unfertilized ovules (Fig. 2) and seed set (Fig. 3) differed among pollination treatments. The unfertilized ovules following self-pollination only accounted for 0.033±0.011 (mean ± SE) of the total ovules, but it was significantly higher than the other four pollination treatments (*P*<0.05, Fig 2). The seed set after self-pollination was 0.402±0.031and significantly lower than after cross-pollination (0.587±0.026: t = 5.78, df = 300, *P*<0.01, Fig. 3), which suggested a significant reduction in female reproductive success after self-pollination.

If a post-zygotic mechanism was wholly responsible for the reduction in female fitness with no associated pre-zygotic LSI, all of the ovules in the chase-pollination treatments would be fertilized by the first-arriving self-pollen grains, hence the seed set would be similar to that after self-pollination [24], [28]. However, in our study, the seed set from chase-pollination (0.505±0.030) was higher than from self-pollination when cross-pollen was applied one day after self-pollen (t = 3.15, df = 300, *P*<0.01, Fig. 3). These results support the hypothesis that pre-zygotic LSI occurs in *A. kusnezoffii*. However, if the pre-zygotic LSI prevented all of the self-pollen from fertilizing ovules, the first-arriving self-pollen grains would not be able to fertilize any ovules in the chase-pollination treatment, and the ovules would then be available to the ensuing cross-pollen grains, leading to a seed set similar to that of the cross-pollination treatment. We found that the seed set of the chase-pollination treatment was lower than that of the cross-pollination treatment (t = 2.63, df = 300, *P*<0.01, Fig. 3), indicating that the first-arriving self-pollen grains did fertilize some but not all of the ovules. Therefore, *A kusnezoffii* presents a partial pre-zygotic LSI, with a rejection of part of the self-pollen in the ovary. Besides, self pollen can disable ovules through affecting the development of the embryo sac rather than fertilizing the ovules [7], [9]. Taking such mechanisms into account, the chase experiment may underestimate the importance of pre-zygotic LSI.

The unfertilized ovules following self-pollination was only accounted for 0.033 of the total ovules (Fig. 2). Therefore, the pre-zygotic self-pollen rejection make a minimal contribution to the reduced seed set after self-pollination (0.402±0.031, a 32% reduction compared to cross-pollination), and the post-zygotic processes have a relatively large influence. However, in the chase-pollination the late-arriving cross-pollen significantly increased the seed set (0.505±0.030), thus it seems much more than 3.3% of the ovules were available for fertilization by cross-pollen in the chase-pollinated fruits. One possible explanation is that although self-pollen tube can grow into ovaries in 12 hours after hand-pollination [33], not all of the pollen tubes immediately released sperms and garnered the ovules; as a consequence, a higher-than-expected proportion of the ovules were still available when the cross-pollen tubes arrived one day later. Apparently further work is needed to confirm this conjecture.

### Early-acting ID and/or LSI

**Figure 4:**
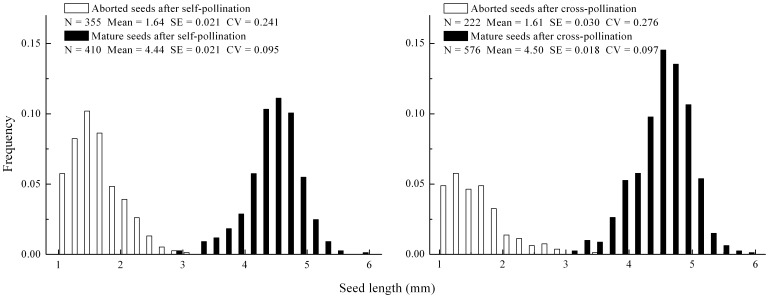
Seed length distribution of mature and aborted seeds after self-pollination in *Aconitum kusnezoffii*. Thirteen selfed fruits (a) and crossed fruits (b) were chosen randomly, and all of their aborted and mature seeds were measured.

**Figure 5:**
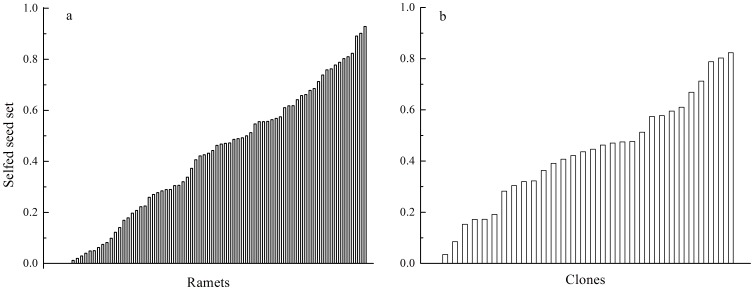
Variation in seed set (one selfed fruit for each of 76 ramets) following self-pollination in *Aconitum kusnezoffii* shows a continuous, widespread distribution.

Variation in seed size and selfed seed set is usually regarded as an important factor that discriminates between early-acting ID and post-zygotic LSI. We found that following self-pollination, aborted seeds varied widely and continuously in length (Fig. 4a). The coefficient of variation (CV) of the length of aborted selfed seeds (CV = 0.241) was larger than that of mature selfed seeds (CV = 0.095, t =  −9.62, df = 12, *P*<0.01). Although the proportion of aborted seeds following cross-pollination (28%) was smaller than self-pollination (46%), the seed-size distribution of aborted and mature seeds was rather similar in both cases (Fig. 4b). Selfed seed set as estimated by measuring the one selfed fruit from each of 76 sampled ramets varied extensively and continuously, from 0–93% at the ramet-level (Fig. 5a) and from 0–82% at the clone-level (Fig. 5b).

Early-acting ID generally involves the expression of recessive deleterious genes at multiple loci and hence often leads to a wide range of aborted seed sizes [5], [12], [27], [28] and/or a wide range of selfed seed sets [12], [17], [27]. While LSI is usually thought to be controlled by a few genes, so the distribution of aborted seed sizes and seed set after self-pollination should follow a clumped pattern. Therefore, the above results imply that abortion occurred at different stages during seed development, rather than at a uniform stage, which supported the idea that early-acting ID was operating in *A. kusnezoffii* and excluded the possibility of a complete post-zygotic LSI. In *Vaccinium corymbosum*, Krebs & Hancock (1991) found that the selfed seed set exhibited a great deal of variation, which was thought to be consistent with the expectations of the early-acting ID hypothesis [17].

**Figure 6:**
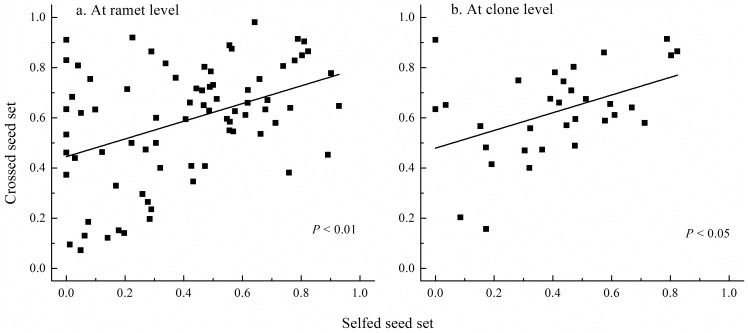
Correlation between the selfed and crossed seed sets in *Aconitum kusnezoffii* at the ramet level (a) and clone level (b).

Furthermore, selfed and crossed seed sets correlated positively at both the ramet- (correlation coefficient = 0.41, t = 3.91, df = 74, *P*<0.01, Fig. 6a) and clone-level (correlation coefficient = 0.45, t = 2.73, df = 30, *P*<0.05, Fig. 6b). These results were also consistent with the early-acting ID hypothesis but not with self-incompatibility, because with early-acting ID, the genotype with many deleterious alleles will experience poor seed set following both self- and cross-fertilization [24], so that self- and cross-pollination of pairs of flowers on individual plants should generate a significant positive correlation in seed sets among plants [17], [27]. In contrast, with LSI, the reduction in the selfed seed set can be attributed to the paternal parent carrying the same S alleles as the maternal parent [1], [12], [35], hence a correlation between selfed and crossed seed sets is not expected [24]. The positive correlations between self- and cross-fertility were also shown in several *Vaccinium* species, supporting early-acting ID hypothesis [17], [27]. However, environmental heterogeneity among the 76 ramets cannot be ruled out in present study as a confounding factor causing a spurious positive correlation between the selfed and crossed seed sets.

### Summary

We observed reduction of seed set following self-pollination in *A. kusnezoffii*, and differentiated the processes leading to such reduction. The results excluded the possibility of post-zygotic LSI. A partial pre-zygotic LSI was supported by the chase-experiment; and the early-acting ID was supported by the continuous distribution of the size of aborted selfed seed and selfed seed set, and the correlation between selfed and crossed seed sets. In summary, both partial pre-zygotic LSI and early-acting ID were suggested to contribute to the reduction in selfed seed set in *A. kusnezoffii*, with pre-zygotic LSI rejecting part of the self-pollen and early-acting ID aborting part of the self-fertilized seeds, whereas no signature of post-zygotic LSI was found.
